# Effects of the Urease Concentration and Calcium Source on Enzyme-Induced Carbonate Precipitation for Lead Remediation

**DOI:** 10.3389/fchem.2022.892090

**Published:** 2022-04-27

**Authors:** Lin Wang, Wen-Chieh Cheng, Zhong-Fei Xue, Wenle Hu

**Affiliations:** ^1^ School of Civil Engineering, Xi’an University of Architecture and Technology, Xi’an, China; ^2^ Shaanxi Key Laboratory of Geotechnical and Underground Space Engineering (XAUAT), Xi’an, China

**Keywords:** urease enzyme, carbonate precipitation, heavy metal, urease concentration, calcium source

## Abstract

Heavy metal contamination during the rapid urbanization process in recent decades has notably impacted our fragile environments and threatens human health. However, traditional remediation approaches are considered time-consuming and costly, and the effect sometimes does not meet the requirements expected. The present study conducted test tube experiments to reproduce enzyme-induced carbonate precipitation applied to lead remediation under the effects of urease concentration and a calcium source. Furthermore, the speciation and sequence of the carbonate precipitation were simulated using the Visual MINTEQ software package. The results indicated that higher urease concentrations can assure the availability of CO_3_^2−^ during the enzyme-induced carbonate precipitation (EICP) process toward benefiting carbonate precipitation. The calcium source determines the speciation of carbonate precipitation and subsequently the Pb remediation efficiency. The use of CaO results in the dissolution of Pb(OH)_2_ and, therefore, discharges Pb ions, causing some difficulty in forming the multi-layer structure of carbonate precipitation and degrading Pb remediation. The findings of this study are useful in widening the horizon of applications of the enzyme-induced carbonate precipitation technology to heavy metal remediation.

## Introduction

Heavy metal contamination in the soil and underground water seriously threatens surrounding fragile environments and human health (Kim et al., 2001; Mohan et al., 2006; Bai et al., 2017; Chang et al., 2019; Chen et al., 2022a; Hu et al., 2020; Bai et al., 2021; Hu et al., 2021; Liu et al., 2021; Xue et al., 2021; Hu et al., 2022; Wang et al., 2022). Such heavy metal contamination remediation by the traditional soil flushing measure is deemed time-consuming and costly since heavy metal ions are easily adsorbed by soil particles, and intensive industrial activities further aggravate heavy metal contamination (Yang et al., 2014; An et al., 2019; Kumar and Dwivedi, 2019; Bai et al., 2021; Cheng et al., 2021; Duan et al., 2021; Wei et al., 2021; Yuan et al., 2021; Chen et al., 2022b). In the past few years, various remediation measures, including but not limited to physical, chemical, and biological measures, have been proposed to deal with heavy-metal contamination (Dhami et al., 2013; Shashank et al., 2016; Qian et al., 2017; Rahman et al., 2020; Ahenkorah et al., 2021a; Hu et al., 2021; Jiang et al., 2021; and Xue et al., 2022). Amongst the heavy metal contamination remediation measures, enzyme-induced carbonate precipitation (EICP) is an environmentally friendly, efficient, and sustainable measure. Recently, the EICP technology has been widely applied to calcareous sand reinforcement (Ahenkorah et al., 2021b; Chen et al., 2021; Cui et al., 2021; and Meng et al., 2021), while studies on the remediation of contaminations, including organic and inorganic contaminants using the EICP technology, are remarkably limited (Neupane et al., 2013; Putra et al., 2016; and Moghal et al., 2020a). During the EICP process, urease catalyzes urea hydrolysis toward producing CO_3_^2−^. The obtained CO_3_^2−^ precipitates with a calcium source, resulting in carbonate precipitation (Fisher et al., 2017; and Sun et al., 2021). Eqs 1–4 show the biochemical reactions involving urea hydrolysis driven by urease in the EICP process (Mobley and Hausinger, 1989; Fujita et al., 2010; Achal et al., 2012; Achal et al., 2013; Mitchelf et al., 2013; Mugwar and Harbottle, 2016).

$$CO{\left(NH_{2}\right)}_{2}+H_{2}O\to 2NH_{3}+CO_{2},$$ (1)

$$2NH_{3}+2H_{2}O↔2NH_{4}^{+}+2OH^{-},$$ (2)

$$CO_{2}+2OH^{-}↔HCO_{3}^{-}+OH^{-}↔CO_{3}^{2-}+H_{2}O,$$ (3)

$$Ca^{2+}+CO_{3}^{2-}\to CaCO_{3}↓.$$ (4)

A significant body of research conducted over the last few years has greatly enhanced our understanding of improving the mechanical properties of soil and mitigating dust emissions using the EICP technology. Yuan et al. (2020) studied an improved EICP method by adding organic materials (i.e., skim milk powder and brown sugar) into the urease solution and demonstrated its improvement in the mechanical properties of fine-grained soils. Almajed et al. (2018) conducted a baseline study to evaluate the effect of urease solution components on the precipitated efficiency, and a threshold of the carbonate fraction was further identified through a series of unconfined compression tests. Hamdan and Kavazanjian, (2016) performed wind tunnel tests, and the mitigation of fugitive dust emissions was attained by carbonate precipitation using the EICP technology. Hoang et al., (2019) examined the shearing resistance in sand and silt-sand mixtures using the carbonate precipitation catalyzed by the urease enzyme extracted from plants. These results demonstrate an exciting potential for the use of the EICP technology to achieve soil reinforcement and mitigation of dust emissions.

Recently, it is also attempted as a remediation measure for immobilizing organic and inorganic contaminants. Moghal et al., 2020b evaluated the efficiency of EICP in restricting the migration of heavy metals by using ethylenediaminetetraacetic acid (EDTA) and citric acid as extractants. The results indicated that the retention efficiency of the remediated soil can be improved by 30% in comparison with the unremediated soil. Nam et al. (2016) examined the ability of the *Canavalia ensiformis* extract to catalyze urea to form carbonate precipitation on heavy metals resulting from abandoned mines and employed X-ray diffraction and scanning electron microscopy (SEM) to confirm the precipitation speciation. The findings of this work suggested that such an extract is effective in immobilizing heavy metals and preventing their diffusion into surrounding environments. Although there are limited studies on the immobilization of heavy metals by the EICP approach, higher concentrations of contaminants (i.e., > than 5 mmol/L in solution or 400 mg/kg in soil) have been neglected (Li et al., 2015; Kang and So, 2016; Zhu et al., 2017). The Pb contamination sites correspond to approximately 20% in all heavy metal–contaminated sites in mainland China, while the highest Pb concentration measured near a smelter in the Fujian province was up to 30,430 mg/kg (Duan et al., 2016). Therefore, an efficient, economical measure to remediate the heavy metal–contaminated sites is of great necessity to protect fragile environments. However, commercial and bacteria-derived sources of the urease enzyme are either expensive or cumbersome (Nafisi et al., 2019; Wu et al., 2020). The objectives of this study are as follows: 1) to investigate the effects of the urease concentration and calcium source on the enzyme-induced carbonate precipitation for high concentration Pb remediation through a series of preliminary test tube experiments using the plant-derived urease enzyme as the catalyst and 2) to identify the speciation and sequence of carbonate precipitation using Visual MINTEQ simulations toward revealing the mechanisms affecting the Pb remediation efficiency.

## Materials and Methods

### Urease Enzyme Extraction

*Canavalia ensiformis* was used in this study to prepare the urease enzyme using simple, economical extraction processes, including crushing, sieving, primary centrifugation, and secondary centrifugation (Yuan et al., 2020). The details are as follows: 1) *Canavalia ensiformis* was crushed using the plant grinding machine, thereby sieving with a mesh sieve (apertures = 0.15 mm); 2) the jack bean and ethanol solution were mixed in a ratio of 1:10 (g: ml), and the mixture solution was centrifuged for 30 min and then stored in the refrigerator for 4 h; 3) after that, the solution was centrifuged again for 1 h, and the precipitation of secondary centrifugation was the urease used in this study. It should be noted that the urease enzyme should be stored in a refrigerator at −20°C prior to its use. The urease enzyme is suggested to be used within 24 h after it is derived.

### Urease Activity Tests

At standard conditions (30°C), 1 international unit (IU) of the enzyme activity is the amount of 1.0 μmol catalyzed transformation in 1 min (Mobley and Hausinger, 1989). Furthermore, a modified measure, proposed by Van Paassen (2011), was also used to enhance the accuracy of urease activity measurements. For the test, 1 ml of urease solution was mixed with 9 ml 1.11 mol/L urea, with electrical conductivity (*EC*), measured at 5, 10, and 15 min after mixing at room temperature. The modified measure can be expressed as follows:

$$\text{U}\text{r}\text{e}\text{a}\text{s}\text{e}\;\text{a}\text{c}\text{t}\text{i}\text{v}\text{i}\text{t}\text{y}=\frac{EC_{5}+EC_{10}+EC_{15}}{15}\times 10\times 11.11\left(\text{m}\text{m}\text{o}\text{l}\;\text{u}\text{r}\text{e}\text{a}\;\min^{-1}\right),$$ (5)

where *EC*_5_, *EC*_10_, and *EC*_15_ are electrical conductivities at 5, 10, and 15 min, respectively. The modified electrical conductivity method yielded the urease activity being 99.8 mM Urea min-1, while the Nessler’s reagent colorimetric method yielded a urease activity of 342.7 U/g. The former reflects the amount of urea hydrolyzed per minute. However, the latter reflects the required amount of enzyme converting 1 μM of the substrate using a specialized unit U/g. For this reason, the two values are not found on the same basis, although the Nessler’s reagent colorimetric method adopted in the present work can describe the urease activity in a more straightforward manner. On the other hand, the NH_4_^+^ concentration was measured *via* a combination of the Nessler’s reagent and spectrophotometer, which aims to describe the degree of urea hydrolysis.

### Test Tube Experiments

The effects of urease concentration and the calcium source on Pb remediation were investigated through a series of test tube experiments. Three urease concentrations (3 g/L, 6 g/L, and 9 g/L) and three calcium sources (CaCl_2_, Ca(CH_3_COO)_2_, and CaO) were adopted in the test tube experiments. The calcium source concentration used in the test tube experiments is 0.25 mol/L, while the urea concentration is 0.5 mol/L. Lead contaminant (Pb(NO_3_)_2_) concentrations adopted include both the low and high ranges, namely, 5 mmol/L, 10 mmol/L, 30 mmol/L, 40 mmol/L, and 50 mmol/L. The solution applied to the test tube experiments consisted of distilled water, urea, Pb(NO_3_)_2_, CaCl_2_, and the urease enzyme. The preparation of the solution is depicted in Supplementary Figure S1. Catalyzing urea hydrolysis was handled at an indoor temperature of about 26°C and was conducted for a 48-h period, following inoculation. The measurements of pH, NH_4_^+^ concentration, and remaining Pb^2+^ concentration were conducted after 48 h. It is widely accepted that NH_4_^+^ and OH^−^ are the products of urea hydrolysis using the EICP technology, and they determine whether comprehensive urea hydrolysis is attained. The degree of urea hydrolysis not only dominates the amount of carbonate precipitation but also reflects the remaining Pb^2+^ concentration and remediation efficiency. The more the Pb^2+^ precipitated with the carbonate, the lesser will be the remaining Pb^2+^ and the higher the remediation efficiency. Prior to the measurement of the NH_4_^+^ concentration, the mixed solution was centrifuged and acidified to a pH lower than 2.0 for the sake of NH_4_^+^ measurement. Furthermore, the mixed solution was diluted 100–500 times, allowing the measurement to be undertaken. It was worth noting that all the precipitations were separated from the solution through the vacuum filtration method at the end of the tests and dried at 30°C for 3 days before being subjected to mass measurement (Keykha et al., 2018). All aforementioned tests had three biological replicates with coefficient of variance (COV) **<** 10%.

### Visual MINTEQ Simulation

Given that the test tube experiments are not capable of providing the speciation and sequence of carbonate precipitation, it was simulated using the Visual MINTEQ software package. Here, urea hydrolysis was modeled as the initial condition about NH_4_^+^ and CO_3_^2−^ with a stoichiometric ratio of 2:1 (Gat et al., 2017). The simulation for the evolution of the precipitation speciation was determined by the degrees of urea hydrolysis that require the inputs of NH_4_^+^ and CO_3_^2−^ concentrations toward differentiating the abiotic precipitation from the biotic precipitation that directly plays roles in urea hydrolysis (Xue et al., 2022).

## Results

### Relationship of Urease Concentration versus Lead Remediation Efficiency

**FIGURE 1:**
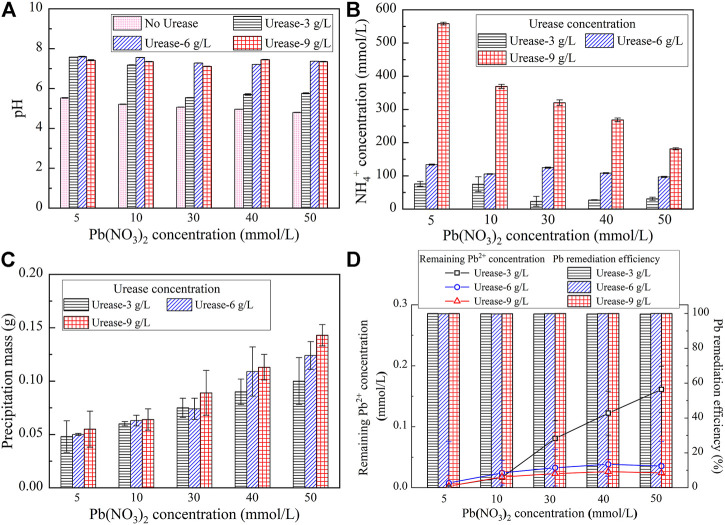
**(A)** Relationship of pH versus Pb(NO_3_)_2_ concentration; **(B)** Relationship of the NH_4_^+^ concentration versus Pb(NO_3_)_2_ concentration; **(C)** Relationship of the precipitation mass versus Pb(NO_3_)_2_ concentration; and **(D)** Relationship of the Pb^2+^ concentration and Pb remediation efficiency versus Pb(NO_3_)_2_ concentration.

It is widely accepted that ammonium (NH_4_^+^) and hydroxide (OH^−^) ions are the products of urea hydrolysis using the EICP technology (Eqs 1–3), and therefore, the NH_4_^+^ concentration and pH are considered appropriate in determining whether comprehensive urea hydrolysis is attained. Its measurements were conducted during the EICP process. The variations of NH_4_^+^ concentration and pH when subjected to no urease and urease at concentrations ranging from 3 g/L to 9 g/L are shown in Figures 1A,B. Prior to introducing urease, the pH is always lower than 6 and appears insensitive to the concentration of Pb(NO_3_)_2_. However, the pH increases and surpasses 6 while catalyzing urea hydrolysis using the urease enzyme. Furthermore, the pH is generally increased with the increase of the urease concentration, and for urease at 3 g/L, there is a decrease when subjected to Pb(NO_3_)_2_ at 30 mmol/L (see Figure 1A). On the other hand, the urease is also on the way to catalyze urea hydrolysis toward producing NH_4_^+^ (see Figure 1B). Furthermore, the NH_4_^+^ concentration is generally decreased with the increase of the Pb(NO_3_)_2_ concentration. A significant drop in the Pb(NO_3_)_2_ concentration is also present when Pb(NO_3_)_2_ is at 30 mmol/L. Such a drop is not significant when subjected to higher urease concentrations (e.g. > 3 g/L). The variations of the measured precipitation mass when subjected to urease at 3 g/L, 6 g/L, and 9 g/L are shown in Figure 1C. It is clear that the precipitation mass is increased with the increase of the urease concentration and its maximum being 0.14 g, resulting from the urease at 9 g/L, presents when the Pb(NO_3_)_2_ concentration is at 50 mmol/L. Furthermore, the produced precipitation mass is generally increased with the increase of the Pb(NO_3_)_2_ concentration.

To assess the effectiveness of Pb remediation, a remediation efficiency defined as a ratio of the remaining Pb^2+^ concentration to the initial Pb^2+^ concentration was used in the present study. The lower the remaining Pb^2+^ concentration, the higher is the Pb^2+^ remediation efficiency. The variations of the remaining Pb^2+^ concentration and Pb remediation efficiency when subjected to urease at 3 g/L, 6 g/L, and 9 g/L, respectively, are shown in Figure 1D. The relationship of the remaining Pb^2+^ concentration versus Pb(NO_3_)_2_ concentration for the urease at 3 g/L lies above that for the urease at 6 g/L which is underlaid by that for the urease at 9 g/L. These results show that the higher the urease concentration, the lower is the remaining Pb^2+^ concentration. By measuring the remaining Pb ion concentration, the remediation efficiency against different urease concentrations is measured to be higher than 99.5%. Its maximum presents when the urease is at 9 g/L and reaches approximately 100%.

### Relationship of Calcium Source Versus Lead Remediation Efficiency

**FIGURE 2:**
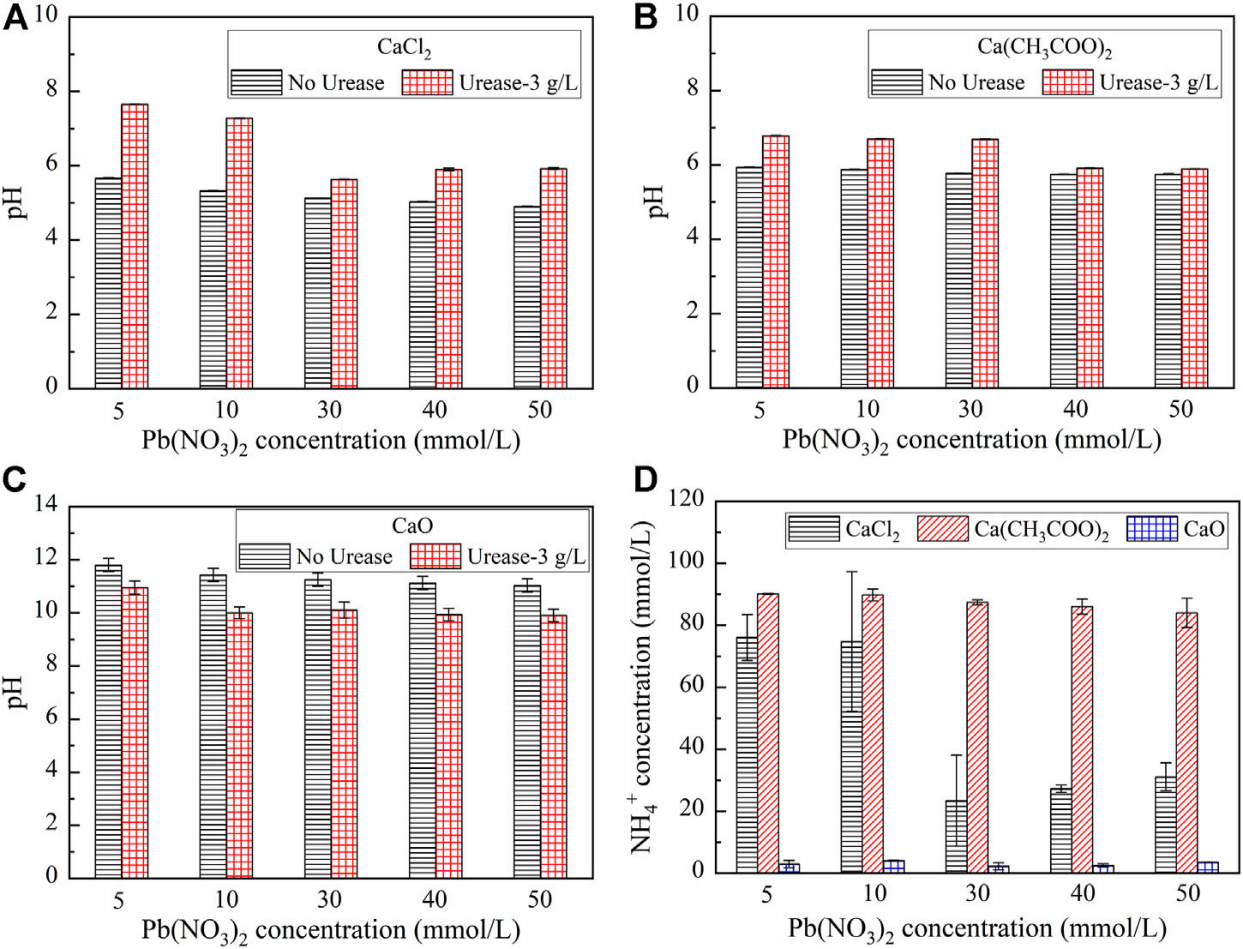
Relationship of pH and NH_4_^+^ concentrations versus the Pb(NO_3_)_2_ concentration considering different calcium sources: **(A)** CaCl_2_; **(B)** Ca(CH_3_COO)_2_; **(C)** CaO; and **(D)** Relationship of the NH_4_^+^ concentration versus Pb(NO_3_)_2_ concentration.

Given that the effect of the calcium source is considered crucial in improving Pb remediation, three calcium sources, including calcium chloride (CaCl_2_), calcium acetate (Ca(CH_3_COO)_2_), and calcium oxide (CaO), were taken into account in the test tube experiments. Despite the Pb remediation efficiency being approximately 100% at 9 g/L, an efficiency being higher than 99.5% was derived at 3 g/L. The efficiency of 3 g/L was, therefore, adopted in the following analysis for the sake of economy. The variations of pH and NH_4_^+^ concentrations when subjected to CaCl_2_, Ca(CH_3_COO)_2_, and CaO are shown in Figure 2. The pH, while catalyzing urea hydrolysis using urease, is always in excess of that prior to the urea hydrolysis except the case of calcium oxide (see Figures 2A–C). Furthermore, the pH is decreased with the increase of the Pb(NO_3_)_2_ concentration. Moreover, for the same Pb(NO_3_)_2_ concentration, the NH_4_^+^ concentration is the highest when subjected to Ca(CH_3_COO)_2_ (see Figure 2D). The NH_4_^+^ concentration is the lowest when subjected to CaO.

**FIGURE 3:**
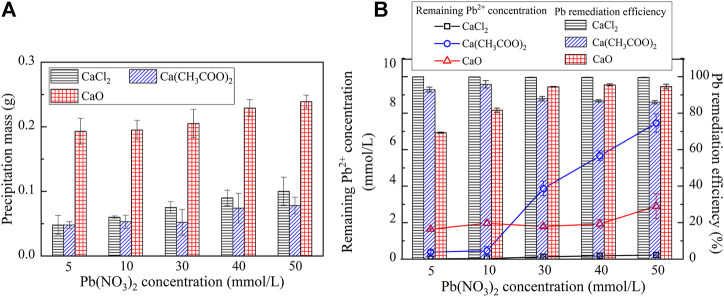
**(A)** Relationship of the precipitation mass versus Pb(NO_3_)_2_ concentration and **(B)** Relationship of the Pb^2+^ concentration and remediation efficiency versus Pb(NO_3_)_2_ concentration.

The variations of the measured carbonate precipitation mass when subjected to CaCl_2_, Ca(CH_3_COO)_2_, and CaO are shown in Figure 3A. The results indicate that the carbonate precipitation mass, when subjected to CaO, is much greater than that subjected to CaCl_2_ and Ca(CH_3_COO)_2_, and its maximum reaches 0.23 g. Furthermore, the precipitation mass is generally increased with the increase in the Pb(NO_3_)_2_ concentration. The variations of the remaining Pb^2+^ concentration and Pb remediation efficiency, when subjected to CaCl_2_, Ca(CH_3_COO)_2_, and CaO, are shown in Figure 3B. The relationship of the remaining Pb^2+^ concentration versus Pb(NO_3_)_2_ concentration, when subjected to Ca(CH_3_COO)_2_ and CaO, lies above that subjected to CaCl_2_. The Pb remediation efficiency, when subjected to different calcium sources, performs very well and is generally in excess of 70%. The best remediation efficiency measures at about 100%.

### Visual MINTEQ Simulation

**FIGURE 4:**
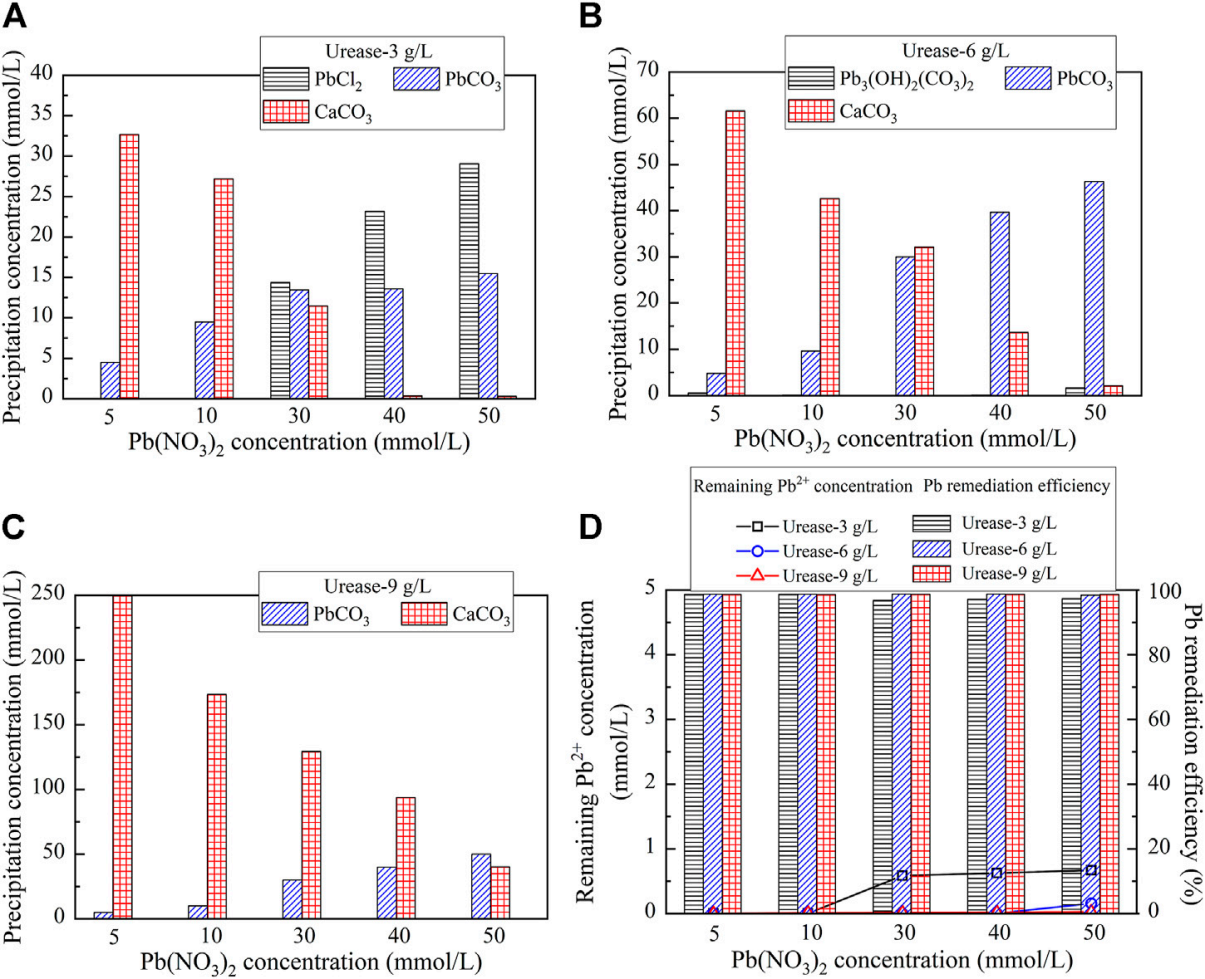
Visual MINTEQ simulation under different urease concentrations: **(A)** 3 g/L; **(B)** 6 g/L; **(C)** 9 g/L; and **(D)** remaining Pb^2+^ concentration and remediation efficiency.

Carbonate precipitations, including abiotic precipitation and biotic precipitation, in the test tube experiments, were reproduced using the Visual MINTEQ software package, investigating its speciation during the EICP process. Understanding the speciation of precipitations is considered of great necessity for revealing the mechanisms affecting Pb remediation. The reproduced carbonate precipitations, when subjected to urease at 3 g/L, 6 g/L, and 9 g/L, are shown in Figure 4. There are four precipitation speciations present, including PbCl_2_, hydrocerrusite (Pb_3_(CO_3_)_2_(OH)_2_), lead carbonate (PbCO_3_), and calcium carbonate (CaCO_3_) (Eqs 4, 6, 7, 8). PbCl_2_ is categorized as abiotic precipitation since it is formed prior to introducing urease, while Pb_3_(CO_3_)_2_(OH)_2_, PbCO_3_, and CaCO_3_ are, therefore, classed as biotic precipitation. Furthermore, accompanied with the increase of the urease concentration, the precipitation speciation starts transforming. For example, PbCl_2_ is transformed to Pb_3_(CO_3_)_2_(OH)_2_ in the first place when the urease concentration is increased from 3 g/L to 6 g/L (see Figures 4A–C). Subsequently, Pb_3_(CO_3_)_2_(OH)_2_ is transformed to PbCO_3_ when the urease concentration is increased from 6 g/L to 9 g/L. Moreover, except CaCO_3_ the concentrations of PbCl_2_, PbCO_3_, and Pb_3_(CO_3_)_2_(OH)_2_ are increased with the increase in the Pb(NO_3_)_2_ concentration. The simulated results indicate that the remediation efficiency against different urease concentrations performs very well and in excess of 99% on average, which is in line with the experimental results (see Figures 1D, 4D).

$$Pb^{2+}+2Cl^{-}\to PbCl_{2}↓,$$ (6)

$$3Pb^{2+}+2CO_{3}^{2-}+2OH^{-}\to Pb_{3}{\left(CO_{3}\right)}_{2}{\left(OH\right)}_{2}↓,$$ (7)

$$Pb^{2+}+CO_{3}^{2-}\to PbCO_{3}↓.$$ (8)

**FIGURE 5:**
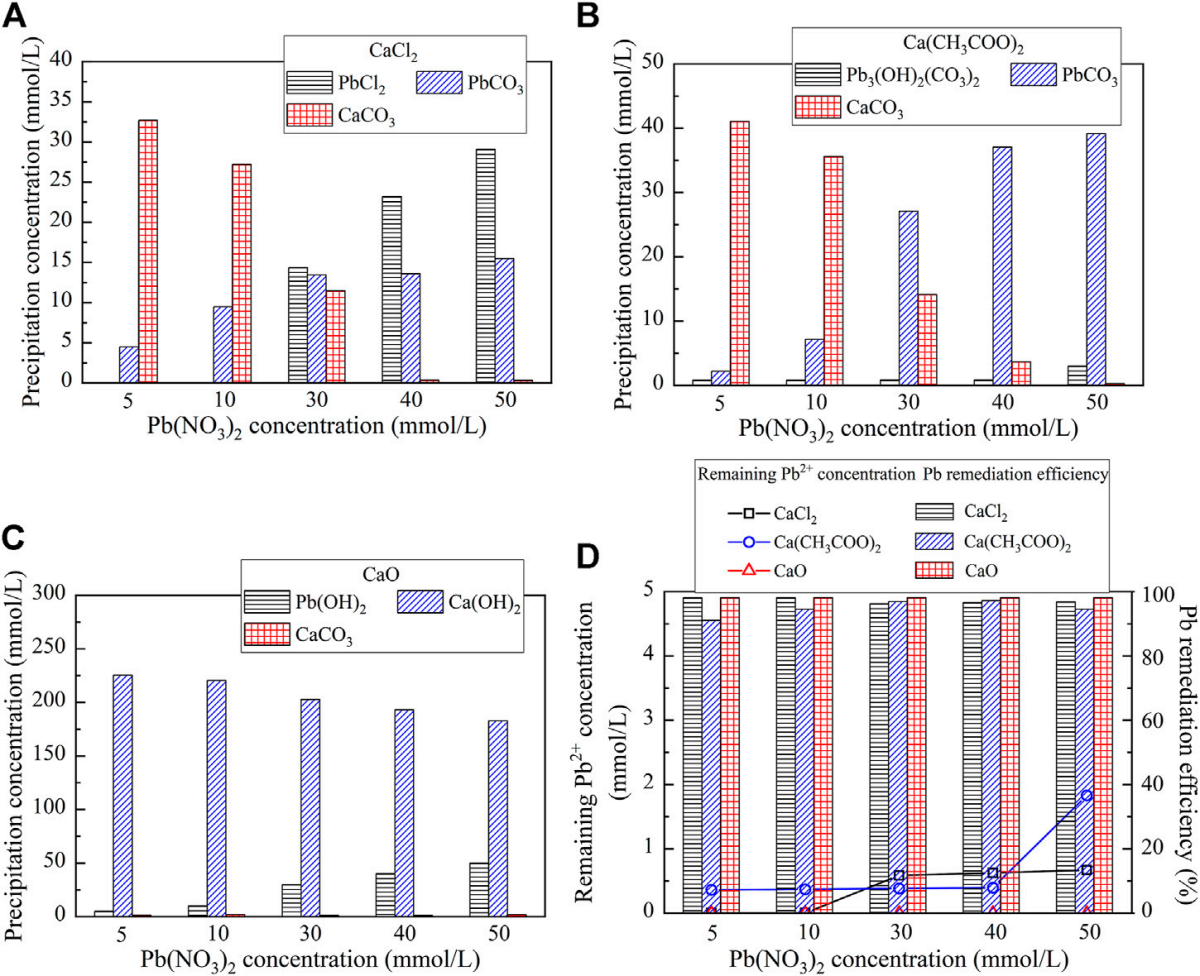
Visual MINTEQ simulation under different calcium sources: **(A)** CaCl_2_; **(B)** Ca(CH_3_COO)_2_; **(C)** CaO; and **(D)** remaining Pb^2+^ concentration and remediation efficiency.

On the other hand, the reproduced carbonate precipitations when subjected to CaCl_2_, Ca(CH_3_COO)_2_, and CaO are shown in Figure 5. Unlike the previous simulation, there are six speciations of carbonate precipitation, including PbCl_2_, Pb(OH)_2_, Pb_3_(CO_3_)_2_(OH)_2_, PbCO_3_, Ca(OH)_2_, and CaCO_3_, when subjected to the effect of the calcium source (Eqs 5–10). PbCl_2_, Pb(OH)_2_, and Ca(OH)_2_ are precipitated before catalyzing urea hydrolysis and are therefore classed as abiotic precipitations, while CaCO_3_, Pb_3_(CO_3_)_2_(OH)_2_, and PbCO_3_ are categorized as biotic precipitations. Furthermore, except CaCO_3_ and Ca(OH)_2,_ the concentrations of PbCl_2_, Pb(OH)_2_, Pb_3_(CO_3_)_2_(OH)_2_, and PbCO_3_ are increased with the increase in the concentration of Pb(NO_3_)_2_ (see Figures 5A–C). Last but not least, the remediation efficiency against different calcium sources surpasses 90% (see Figure 5D). There appears a discrepancy between the simulated results and the experimental ones (see Figures 3B, 5D).

$$Pb+2OH^{-}\to Pb{\left(OH\right)}_{2}↓,$$ (9)

$$CaO+H_{2}O\to Ca{\left(OH\right)}_{2}↓.$$ (10)

## Discussion

### Effect of Urease Concentration

It can be seen from Figure 1A that the pH values for urease at 9 g/L are always higher than those for urease at 3 g/L, indicating a more comprehensive urea hydrolysis. The pH, when subjected to urease at 6 g/L and 9 g/L, respectively, shows a small change when the concentration of Pb(NO_3_)_2_ is increased from 5 mmol/L to 50 mmol/L. In contrast, when subjected to urease at 3 g/L, there appears a decrease in the pH of Pb(NO_3_)_2_ at 30 mmol/L. The lower the urease concentration, the more significant is the effect of Pb ions. A similar decrease can also be seen in Figure 1B; when subjected to urease at 9 g/L and 6 g/L, respectively, the NH_4_^+^ concentration generally decreases with the increase in the Pb(NO_3_)_2_ concentration. However, when subjected to urease at 3 g/L, there appears a NH_4_^+^ concentration drop in Pb(NO_3_)_2_ at 30 mmol/L. These results indicate that the effect of Pb ions can depress urea hydrolysis and becomes more significant when subjected to lower urease concentrations. In the present work, Pb(NO_3_)_2_ at or higher than 30 mmol/L starts depressing urea hydrolysis when subjected to urease at 3 g/L. Urease is essentially protein, and heavy metal ions can badly inactivate it by reacting with its sulfhydryl group (Shaw and Raval, 1961; Chung et al., 2020). Given that the NH_4_^+^ concentration reflects whether comprehensive urea hydrolysis is attained, enhancing our understanding of how the urease activity, when subjected to the effect of Pb ions, affects the production of NH_4_^+^ is considered of great necessity. For this reason, the relation of the urease activity and NH_4_^+^ concentration versus time is explored, as shown in Supplementary Figure S2. It can be observed that the degradation of the urease activity, when subjected to the Pb ions, is not instantaneous (Yuan et al., 2020) and can be further divided into three phases, including the rapid degradation phase, gentle degradation phase, and limited degradation phase, according to the change of the NH_4_^+^ concentration. During the rapid degradation phase, the Pb ions cause the urease activity to decrease very quickly, while the NH_4_^+^ concentration is increased over time, indicating that urease has not been inactivated yet. The urease activity decreases progressively during the gentle degradation phase where the increase of the NH_4_^+^ concentration due to the reduced urease activity is not as significant as before. In the limited degradation phase, the NH_4_^+^ concentration shows a small change, indicating that urease has already been inactivated.

It is well acknowledged that the degradation of the urease activity is accompanied by carbonate precipitation, and the speciation of carbonate precipitation determines whether a fairly good Pb remediation is attained. The following content aims to address how the speciation of carbonate precipitation affects the Pb remediation efficiency. The simulated results indicate that the speciation of carbonate precipitation, when subjected to the urease concentration at 9 g/L, includes PbCO_3_ and CaCO_3_, and they are classed as biotic precipitations. The biotic precipitations are relatively stable compared to the abiotic ones, according to their solubility product (termed K_sp_ hereafter). Their transformation is, therefore, not going to happen (Jiang et al., 2019; Xue et al., 2022). Provided that higher urease concentrations ensure the availability of CO_3_^2−^, a fairly satisfactory Pb remediation can be expected. In contrast, the speciation of carbonate precipitation under the urease concentration at 3 g/L comprises PbCl_2_, CaCO_3_, and PbCO_3_. Two of them (CaCO_3_ and PbCO_3_) are categorized as biotic precipitations and PbCl_2_ as an abiotic one. The Pb remediation efficiencies under 3 g/L and 9 g/L urease concentrations are approximately 100%, although the associated precipitation speciation differs from each other. Given that K_sp_ (PbCl_2_) being 1.6 × 10^–6^ is greater than K_sp_ (PbCO_3_) being 7.4 × 10^–14^, PbCl_2_ is more likely to be dissolved and converted to other chemical substances compared with PbCO_3_. Thus, PbCO_3_ is deemed as a desirable precipitation compound rather than PbCl_2_. To summarize, the effect of Pb ions depresses urea hydrolysis and becomes more significant when subjected to lower urease concentrations. Higher urease concentrations ensure the availability of CO_3_^2−^, contributing to a formation of a relatively stable biotic precipitation.

### Effect of Calcium Source

As the calcium source can affect the speciation of carbonate precipitation, measurements of pH and NH_4_^+^ concentrations can benefit us in enhancing our understanding of the effect of the calcium source on Pb remediation (Jiang et al., 2019). For a given Pb(NO_3_)_2_ concentration, the pH is measured the highest when subjected to CaO, followed by CaCl_2_ (see Figures 2A,C), while the pH is measured the lowest when subjected to Ca(CH_3_COO)_2_ (see Figure 2B). The highest pH derived from CaO appears to indicate the comprehensive urea hydrolysis. In fact, while using CaO as the calcium source, the CaO reacts with H_2_O to form Ca(OH)_2_ toward elevating the value of pH. This is in line with the measurements of pH. The pH reaches a value as high as 12 (see Figure 2C). Notwithstanding this fact, such a high pH depresses the urease activity, resulting in a reduction of the NH_4_^+^ concentration (Mobley and Hausinger, 1989; Neupane et al., 2013; and Almajed et al., 2018) (see Figure 2D). The measured results of the NH_4_^+^ concentration also confirm this statement. On the other hand, the NH_4_^+^ concentration for a given Pb(NO_3_)_2_ concentration is measured the highest under Ca(CH_3_COO)_2_, followed by CaCl_2_. As a result, the calcium source depresses the urease activity by influencing the surrounding pH, resulting in a reduction of the NH_4_^+^ concentration.

The speciation of carbonate precipitation is also explored here to extend the interpretation of the experimental and simulated results. The simulated results indicate that the speciation of the carbonate precipitation, when subjected to the calcium source of CaO, includes Pb(OH)_2_, Ca(OH)_2_, and CaCO_3_ where the former two are classed as abiotic precipitations. Ca(OH)_2_ is formed before Pb(OH)_2_ due to the addition sequence of calcium sources. Given that K_sp_ (Ca(OH)_2_) being 5.5 × 10^–6^ is greater than K_sp_ (Pb(OH)_2_) being 1.2 × 10^–15^, Ca(OH)_2_ tends to transform to Pb(OH)_2_. However, Pb(OH)_2_ is deemed soluble when exposed to alkaline environments (Edwards et al., 1992; Baltpurvins et al., 1996; Vítková et al., 2009; and Wang et al., 2022). When Pb(OH)_2_ is just formed following the aforesaid transformation, and it is also on the way to dissolve, thereby releasing the Pb ions into the solution. This also means a degradation of the Pb remediation. Furthermore, the speciation of carbonate precipitation under the calcium source of Ca(CH_3_COO)_2_ comprises Pb_3_(CO_3_)_2_(OH)_2_, PbCO_3_, and CaCO_3_, and they are classed as biotic precipitations. Under the calcium source of CaCl_2,_ two of them (CaCO_3_ and PbCO_3_) are categorized as biotic precipitations and PbCl_2_ as an abiotic one. The abiotic precipitation compensates for the deficiency of carbonate precipitation caused by the lack of CO_3_^2-^. Therefore, the formation of biotic and abiotic precipitation results in the highest Pb remediation efficiency although the NH_4_^+^ concentration is not the highest under the calcium source of CaCl_2_.

Generally, the higher the precipitation mass, the higher is the remediation efficiency. However, this sometimes causes a misleading interference while assessing the Pb remediation efficiency. In the present work, there appears a discrepancy of Pb remediation between the experimental and simulated results (see Figures 3B, 5D). Despite this fact, the highest precipitation mass against different Pb(NO3)2 concentrations is derived using CaO, and the highest remediation efficiency is, however, derived using CaCl_2_. In light of this, the Pb remediation is not only determined by the precipitation mass but also by the speciation of precipitation. As discussed, the dissolution of Pb(OH)_2_, while using CaO, releases free Pb ions and subsequently degrades Pb remediation, which has been neglected in the simulation. This is considered as the main cause leading to the discrepancy between the experimental and simulated results.

### Mechanisms Affecting Lead Remediation

**FIGURE 6:**
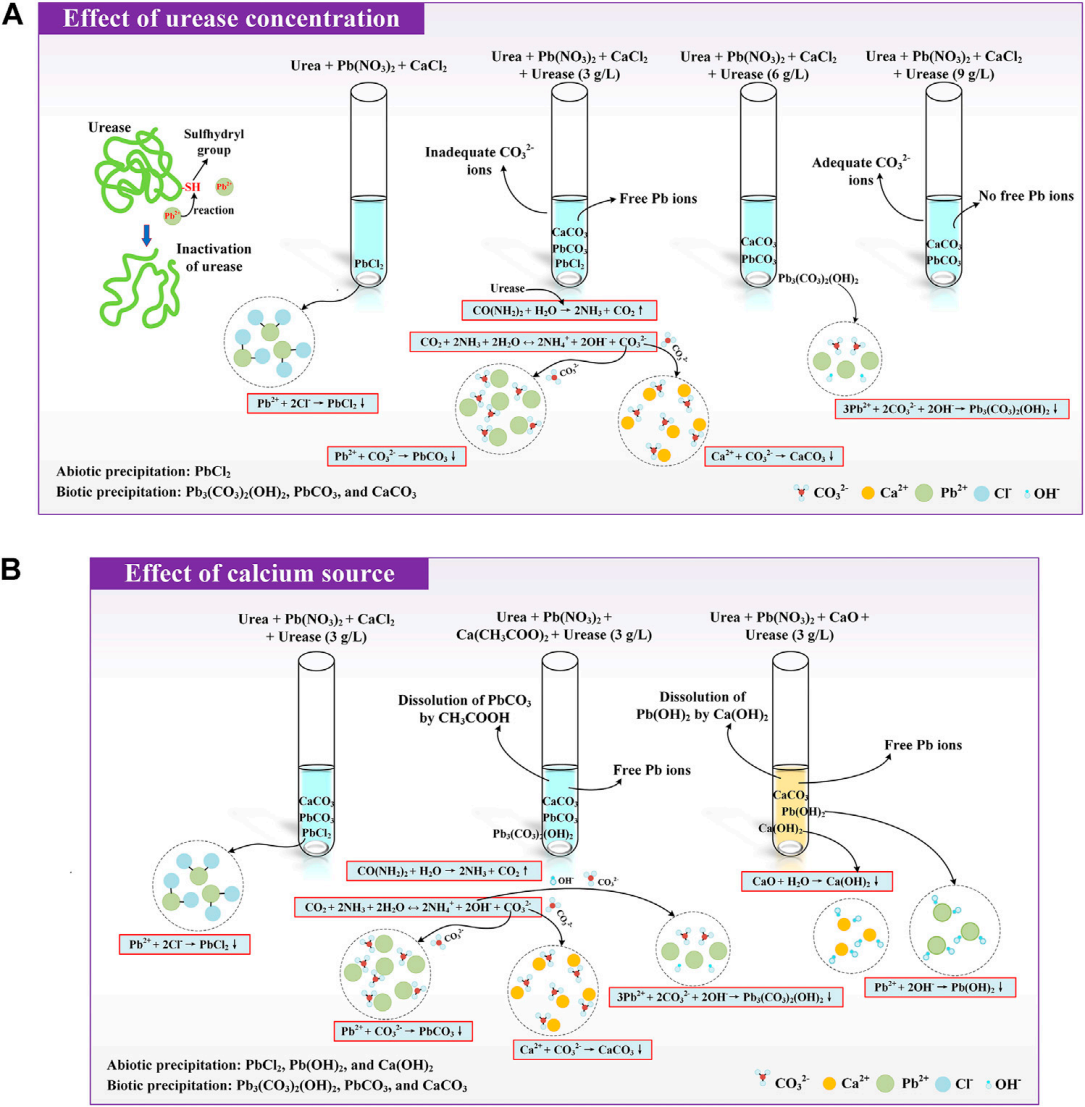
**(A)** Schematic illustration of the effect of urease concentration on Pb remediation and **(B)** Schematic illustration of the effect of the calcium source on Pb remediation.

The change of the NH_4_^+^ concentration confirms that the Pb ions notably depress the urease activity, and the effect of the Pb ions becomes more significant when subjected to lower urease concentrations (see Figure 6A). In these circumstances, the availability of CO_3_^2−^ cannot be consistently assured during the EICP process, thereby degrading Pb remediation. These results indicate that higher urease concentrations not only counterbalance the effect of the Pb ions but also assure the availability of CO_3_^2−^ toward securing a formation of the relatively stable biotic precipitation. On the other hand, the highest pH is derived using CaO, most likely because of the elevation of pH by Ca(OH)_2_. The urease activity is depressed accordingly, and when exposed to alkaline environments, the dissolution of Pb(OH)_2_ releases Pb ions to degrade Pb remediation (see Figure 6B). The highest Pb remediation is attained using CaCl_2_ where the pH is measured in the second place. In short, a combination of higher urease concentrations and an appropriate calcium source may be used to prevent the degradation of Pb remediation.

**FIGURE 7:**
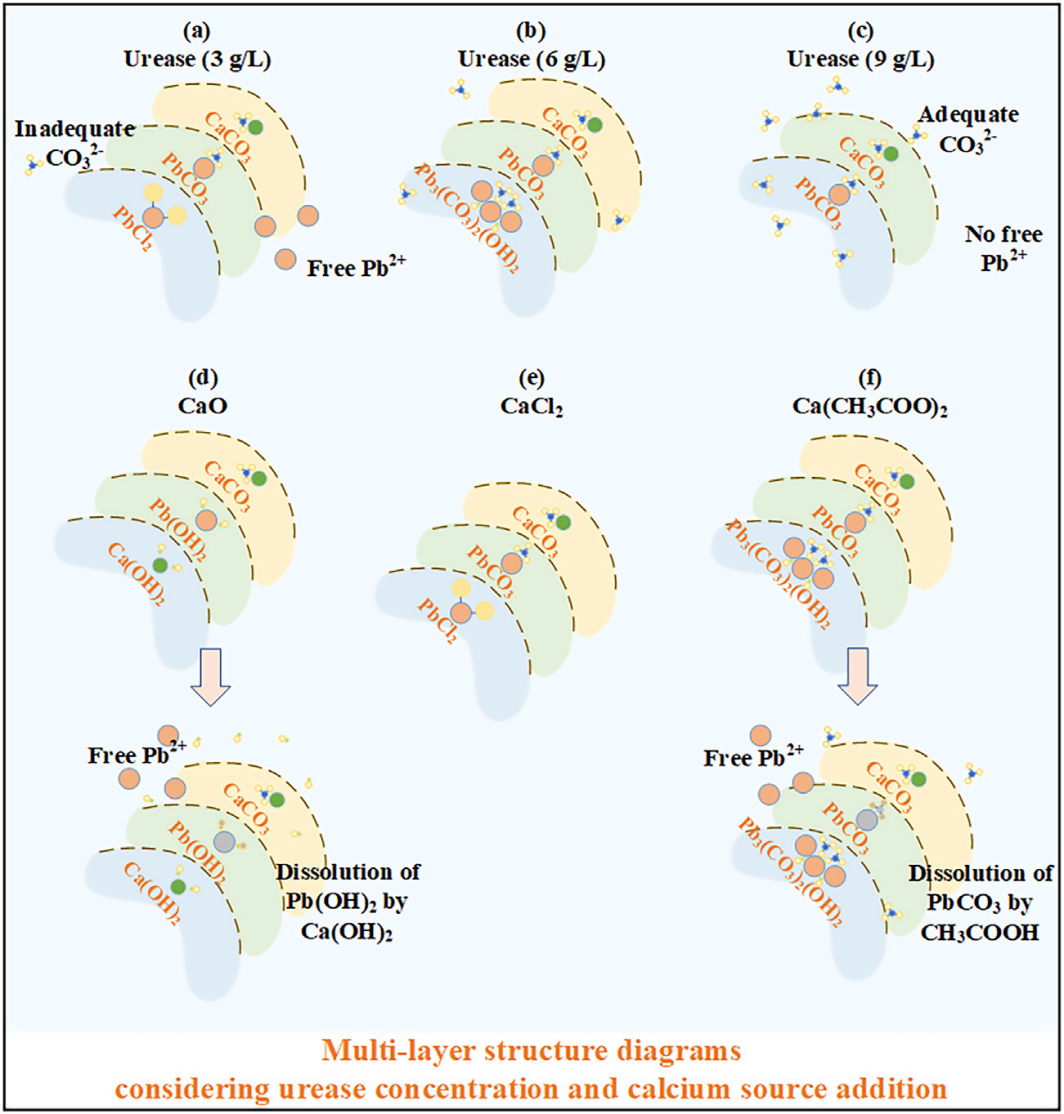
Schematic illustration of the multi-layer structure of carbonate precipitation: **(A)** urease at 3 g/L; **(B)** urease at 6 g/L; **(C)** urease at 9 g/L; **(D)** CaO; **(E)** CaCl_2_; and **(F)** Ca(CH_3_COO)_2._

In addition to the effects of the urease concentration and calcium source, the precipitation mass also has implications on Pb remediation. The precipitation mass under the urease concentration at 9 g/L is higher than that under the urease concentration at 3 g/L, making the discrepancy in Pb remediation efficiency negligible. Meanwhile, the highest precipitation mass against different calcium sources is attained using CaO, and the highest Pb remediation is, however, attained using CaCl_2_. These results confirm that Pb remediation is not only determined by the precipitation mass but also by other influencing factors (e.g., speciation and sequence of carbonate precipitation). When subjected to the urease concentration at 3 g/L, PbCl_2_ first precipitates to form the inner layer of a multi-layer structure, followed by PbCO_3_ and CaCO_3_ to form its outer layer (see Figures 7A–C). The multi-layer structure prevents the migration of Pb ions, although a small number of free Pb ions is released as a result of the inadequacy of CO_3_^2−^. In contrast, the multi-layer structure of carbonate precipitation under urease at 9 g/L can be recognized as PbCO_3_ and CaCO_3_ forming the inner and outer layers, respectively, thereby preventing the migration of Pb ions. On the other hand, Pb_3_(CO_3_)_2_(OH)_2_, when subjected to Ca(CH_3_COO)_2_, precipitates in the first place, followed by PbCO_3_ and CaCO_3_ (see Figures 7D–F). When subjected to CaO, the carbonate precipitation sequence can be sorted as Ca(OH)_2_, Pb(OH)_2_, and CaCO_3_. However, Pb(OH)_2_ is dissolved in alkaline conditions resulting from Ca(OH)_2_, and Pb ions are released, degrading Pb remediation (Edwards et al., 1992; Baltpurvins et al., 1996; Vítková et al., 2009). Moreover, PbCl_2_ under CaCl_2_ first precipitates, followed by PbCO_3_ and CaCO_3_, meaning that a multi-layer structure of carbonate precipitation is developed, and PbCl_2_ forms the inner layer, covered by the two outer layers, including PbCO_3_ and CaCO_3_. The multi-layer structure of the carbonate precipitation encapsulates the Pb ions and causes some difficulty for the Pb ions to migrate, stabilizing the Pb ions and preventing the degradation of Pb remediation. The multi-layer structure of carbonate precipitation, when subjected to CaO, would have been more effective in stabilizing the Pb ions if the dissolution of Pb(OH)_2_ had not happened.

On the whole, Pb ions depress the urease activity, and the effect of Pb ions turns into a more pronounced contributor when subjected to lower urease concentrations. Higher urease concentrations can consistently assure the availability of CO_3_^2−^ during the EICP process. The use of a calcium source can affect the speciation of carbonate precipitation. In some cases, an inappropriate calcium source can cause difficulty in developing the multi-layer structure of carbonate precipitation, thereby degrading Pb remediation. The use of higher urease concentrations and an appropriate calcium source can prevent the degradation of Pb remediation. Furthermore, Pb remediation is not only determined by the precipitation mass but also by other influencing factors. The precipitation sequence plays a role in the formation of the multi-layer structure of carbonate precipitation. The multi-layer structure capsulizes Pb ions and, therefore, prevents their migration, securing the Pb remediation efficiency. The robustness of the multi-layer structure of carbonate precipitation cannot be quantitatively assessed by a micro-structural analysis but by the speciation analysis of Pb^2+^. Details about the results of the micro-structural and speciation analyses are not within the scope of the present work and would be discussed in another article.

## Conclusion

This article has investigated the effects of the urease concentration and a calcium source on Pb remediation. The speciation and sequence of carbonate precipitation have been explored to highlight the mechanisms leading to the degradation of Pb remediation. Based on the results and discussion, some main conclusions can be drawn as follows:

1) The NH_4_^+^ concentration presents good correspondence with the urease concentration. As indicated by the NH_4_^+^ concentration, the Pb ions depress the urease activity, and the effect of Pb ions becomes more significant when provided with lower urease concentrations. Higher urease concentrations can assure the availability of CO_3_^2−^ during the EICP process toward benefiting carbonate precipitation.
2) The calcium source, in fact, determines the speciation of carbonate precipitation and subsequently the Pb remediation efficiency. The Pb remediation efficiency is not only determined by the precipitation mass but also by the other influencing factors (e.g., precipitation sequence). The dissolution of Pb(OH)_2_, when subjected to CaO, has been neglected in the Visual MINTEQ simulation, causing a discrepancy of Pb remediation between the experimental and simulated results. In the present study, the highest Pb remediation efficiency is attained using CaCl_2_. The abiotic precipitation compensates for the deficiency of carbonate precipitation caused by the lack of CO_3_^2−^.
3) The use of CaO results in the dissolution of Pb(OH)_2_ and, therefore, releases Pb ions, causing some difficulty in forming the multi-layer structure of carbonate precipitation and degrading Pb remediation. Given that PbCl_2_ is precipitated first, followed by PbCO_3_ and CaCO_3_, when subjected to CaCl_2_, the multi-layer structure of carbonate precipitation capsulizes the Pb ions and prevents their migration, securing Pb remediation. The present work highlights the exciting potential of applying the EICP technology to Pb ion removal. Further work of stabilizing Pb ions in the contaminated sites by the EICP technology is ongoing and would be discussed in another article.

## Data Availability

The original contributions presented in the study are included in the article/Supplementary Material, further inquiries can be directed to the corresponding author.
