# Supplementary material for: Effects of the Urease Concentration and Calcium Source on Enzyme-Induced Carbonate Precipitation for Lead Remediation
Source: Front Chem. 2022 Apr 27;10:892090. doi: 10.3389/fchem.2022.892090 (PMC9118015; doi:10.3389/fchem.2022.892090)
Supplement: Supplementary file 1 [file DataSheet1.docx]

Supplementary Material

**Supplementary Fig. S1** Schematic illustration of the flowchart applied to the test tube experiments


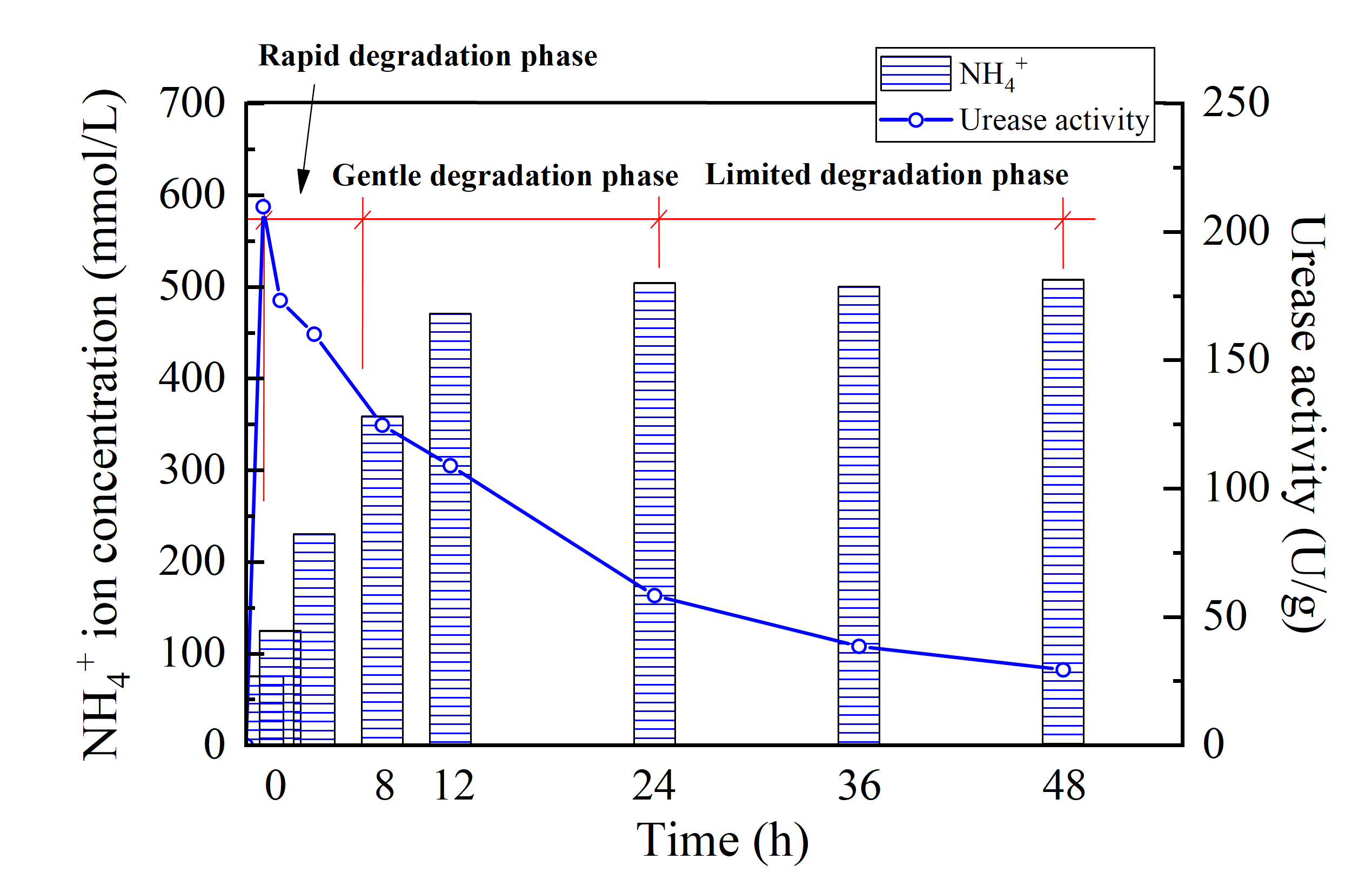


**Supplementary Fig. S2** Relationship of NH_4_^+^ ion concentration and urease activity versus time


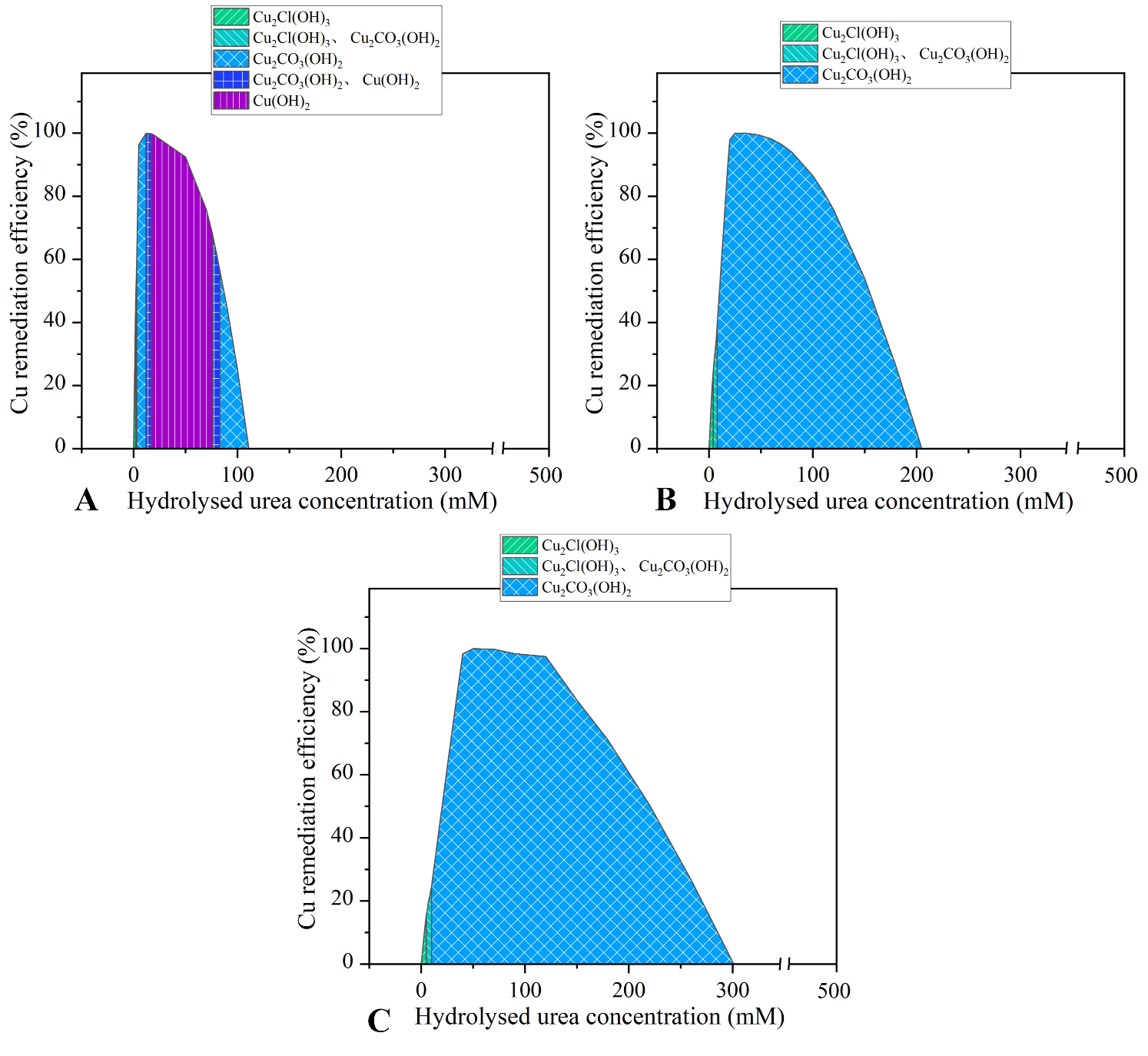


**Supplementary Fig. S3** Simulated relationships of the remediation efficiency versus the hydrolysed urea concentration against Cu(NO_3_)_2_ concentrations (no calcium source addition): (a) 5 mM, (b) 20 mM, and (c) 40 mM


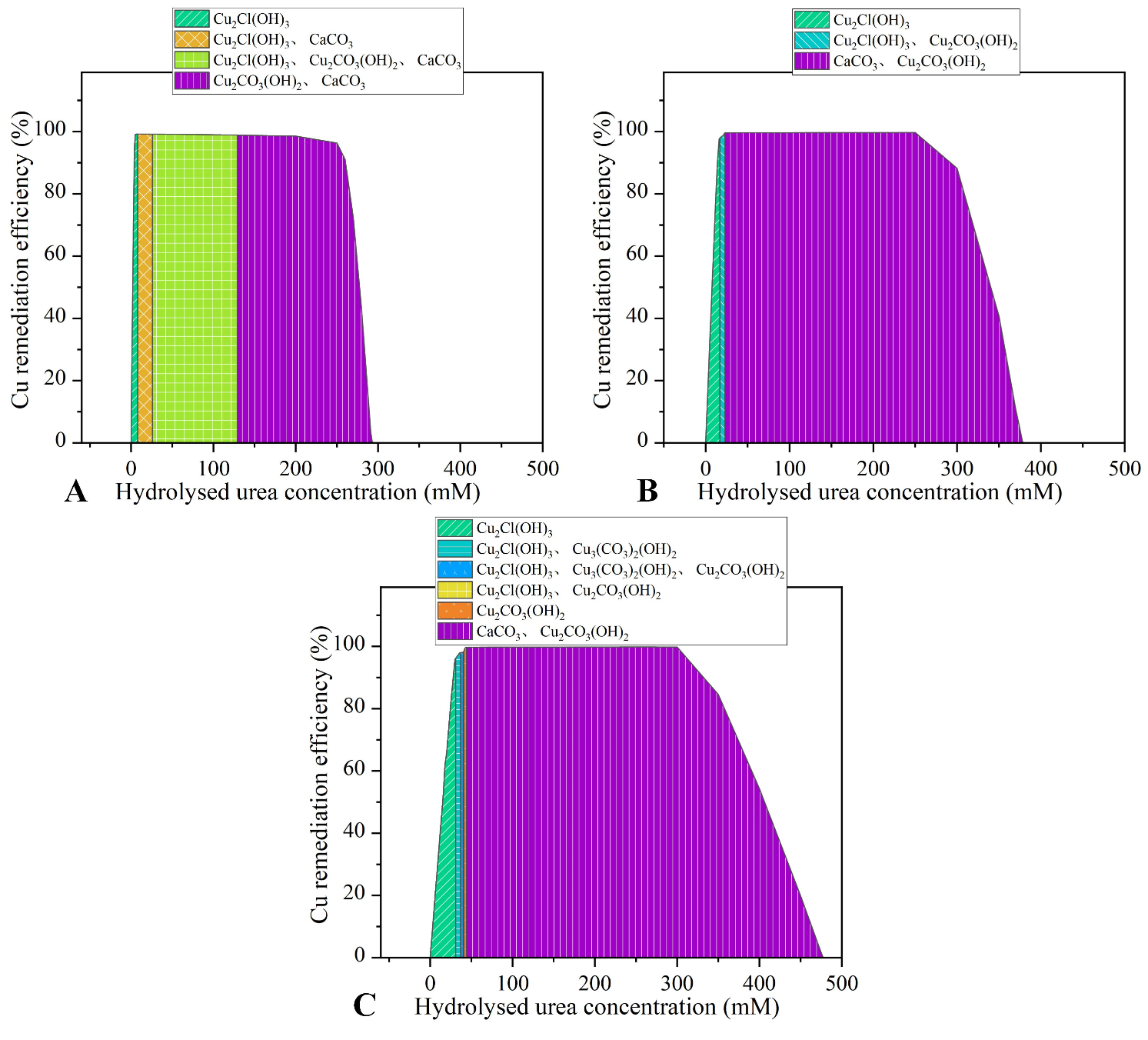


**Supplementary Fig. S4** Simulated relationships of the remediation efficiency versus the hydrolysed urea concentration against Cu(NO_3_)_2_ concentrations (with CaCl_2_ addition): (a) 5 mM, (b) 20 mM, and (c) 40 mM
